# Supplementary material for: Unravelling the Diversity and Abundance of the Red Fox (Vulpes vulpes) Faecal Resistome and the Phenotypic Antibiotic Susceptibility of Indicator Bacteria
Source: Animals (Basel). 2022 Sep 26;12(19):2572. doi: 10.3390/ani12192572 (PMC9558537; doi:10.3390/ani12192572)
Supplement: Supplementary file 1 [file animals-12-02572-s001.zip › animals-1893738-supplementary.pdf]

animals

MDPI

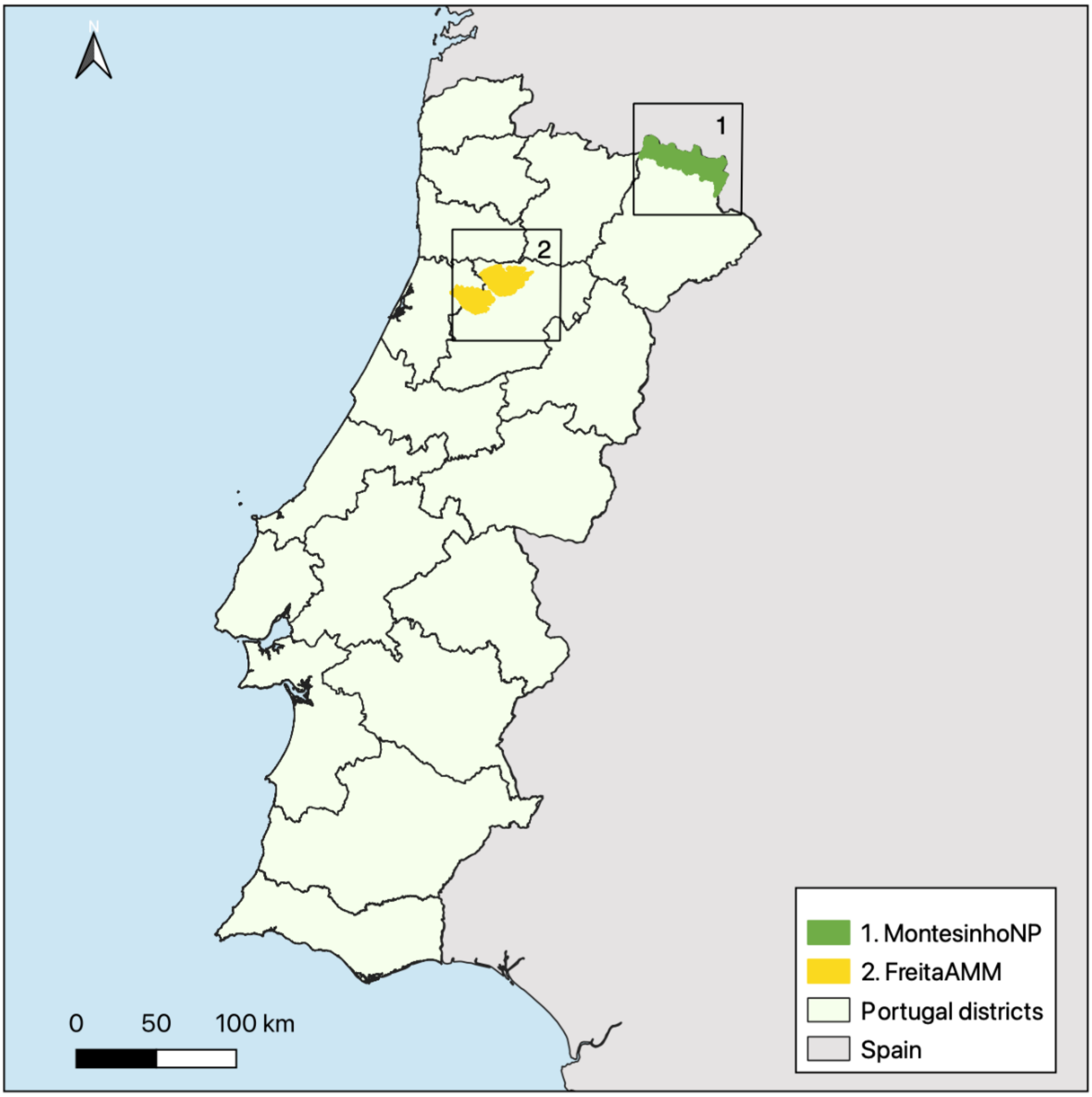

Figure S1. Map of Portugal showing the location of the two sampling sites (MontesinhoNP and FreitaAMM).

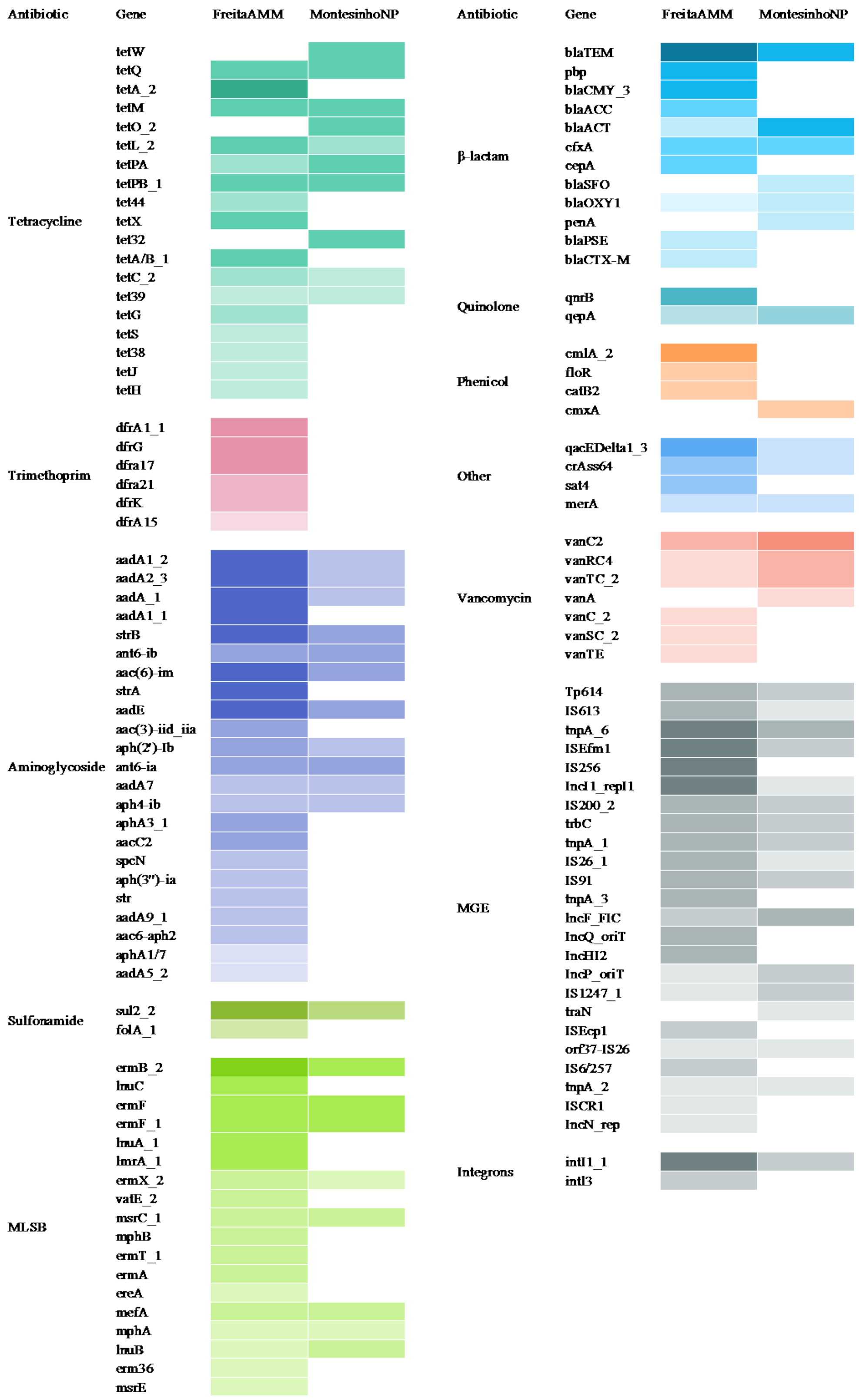

Figure S2. Heatmap representation of the diversity and abundance of ARGs and MGEs identified in red fox faecal samples, according to their geographical localization. Each antibiotic group is represented by a different color and the light to dark color gradient represents low to high abundances, respectively, whereas white represents "not-detected".

Table S1. List of primers selected for the customized array [1].

Name	Forward Primer	Reverse Primer	Target
16S rRNA	GGGTTGCGCTCGTTGC	ATGGYTGTCGTCAGCTCGTG	16S rRNA
Tp614	GGAAATCAACGGCATCCAGTT	CATCCATGCGCTTTTGTCTCT	MGE
IS613	AGGTTCGGACTCAATGCAACA	TTCAGCACATACCGCCTTGAT	MGE
tnpA_6	TGCAGATGGTTTAACCTTGGATATTT	TCGGTTCATCAAACTGCTTCAC	MGE
ISEfm1	AGGTGTCCATGACGTGAAAGTG	TCCTTTGTCCCCTAGGATATTGG	MGE
IS256	CTTGCGCATCATTGGATGATGG	AAGAACGGCTCCAATTAAGCGA	MGE
IncI1_repI1	CGAAAGCCGGACGGCAGAA	TCGTCGTTCCGCCAAGTTCGT	MGE
IS200_2	GCACACCCGATGGAACTGTAAA	TCGGCGGGATCTCCAGAAG	MGE
trbC	CGGYATWCCGSCSACRCTGCG	GCCACCTGYSBGCAGTCMCC	MGE
tnpA_1	GCCGCACTGTCGATTTTTATC	GCGGGATCTGCCACTTCTT	MGE
IS26_1	ATGGATGAAACCTACGTGAAGGTC	CGGTACTTAATCTGTCGGTGTTCA	MGE
IS91	GGATGCCACTGCTGGTCA	ACAGTGGATACAGTATCTGCTGAG	MGE
tnpA_3	GGGCGGGTCGATTGAAA	GTGGGCGGGATCTGCTT	MGE
lncF_FIC	GTGAACTGGCAGATGAGGAAGG	TTCTCCTCGTCGCCAAACTAGAT	MGE
IncQ_oriT	TTCGCGCTCGTTGTTCTTCGAGC	GCCGTTAGGCCAGTTTCTCG	MGE
IncHI2	ATAATGATTCACCGGGGTAG	CTTCAGGCTATCGTTTCG	MGE
IncP_oriT	CAGCCTCGCAGAGCAGGAT	CAGCCGGGCAGGATAGGTGAAGT	MGE
IS1247_1	CGGCCGTCACTGACCAA	TCGGCAGGTTGGTGACG	MGE
traN	GCTTGGCGGTCAGCAATT	TTAGGAATAACAATCGCTACACCTTTA	MGE
ISEcp1	CATGCTCTGCGGTCACTTC	GACGCACCTTCTTGATGACC	MGE
orf37-IS26	GCCGGGTTGTGCAAATAGAC	TGGCAATCTGTCGCTGCTG	MGE
IS6/257	ATATCGTGCCATTGATGCAGAG	ACCATTGCTACCTTCGTTGAAG	MGE
tnpA_2	CCGATCACGGAAAGCTCAAG	GGCTCGCATGACTTCGAATC	MGE
ISCR1	ATGGTTTCATGCGGGTT	CTGAGGGTGTGAGCGAG	MGE
IncN_rep	AGTTCACCACCTACTCGCTCCG	CAAGTTCTTCTGTTGGGATTCCG	MGE
intI1_1	CGAACGAGTGGCGGAGGGTG	TACCCGAGAGCTTGGCACCCA	Integrons
intl3	CAGGTGCTGGGCATGGA	CCTGGGCAGCATCACCA	Integrons
tetW	ATGAACATTCCCACCGTTATCTTT	ATATCGGCGGAGAGCTTATCC	Tetracycline
tetQ	CGCCTCAGAAGTAAGTTCATACACTAAG	TCGTTCATGCGGATATTATCAGAAT	Tetracycline
tetA_2	CTCACCAGCCTGACCTCGAT	CACGTTGTTATAGAAGCCGCATAG	Tetracycline
tetM	GGAGCGATTACAGAATTAGGAAGC	TCCATATGTCCTGGCGTGTC	Tetracycline
tetO_2	CAACATTAACGGAAAGTTTATTGTATACCA	TTGACGCTCCAAATTCATTGTATC	Tetracycline
tetL_2	ATGGTTGTAGTTGCGCGCTATAT	ATCGCTGGACCGACTCCTT	Tetracycline
tetPA	GGAAACCTTAGTTCAGTGACTTGG	CCCATTTAACCACGCACTGAA	Tetracycline
tetPB_1	TGGGCGACAGTAGGCTTAGAA	TGACCCTACTGAAACATTAGAAA-TATACCT	Tetracycline
tet44	CTCATGTAGATGCAGGAAAGACG	GTAACTGCTGCCTGAATTGTGA	Tetracycline
tetX	AAATTTGTTACCGACACGGAAGTT	CATAGCTGAAAAAATCCAGGACAGTT	Tetracycline
tet32	CCATTACTTCGGACAACGGTAGA	CAATCTCTGTGAGGGCATTTAACA	Tetracycline
tetA/B_1	AGTGCGCTTTGGATGCTGTA	AGCCCCAGTAGCTCCTGTGA	Tetracycline
tetC_2	ACTGGTAAGGTAAACGCCATTGTC	ATGCATAAACCAGCCATTGAGTAAG	Tetracycline
tet39	TATAGCGGGTCCGGTAATAGGTG	CCATAACGATCCTGCCCATAGATAAC	Tetracycline
tetG	TCGCGTTCCTGCTTGCC	CCGCGAGCGACAAACCA	Tetracycline
tetS	TTAAGGACAAACTTTCTGACGACATC	TGTCTCCCATTGTTCTGGTTCA	Tetracycline
tet38	AAGCGACATTAGCCGGTTTAG	CTGCTCGTACTTAAGCCAAGG	Tetracycline
tetJ	CAGCGCCCATACGCCATTTA	CCTACTTCAGTAGTGTGCCAAGC	Tetracycline
tetH	TTTGGGTCATCTTACCAGCATTAA	TTGCGCATTATCATCGACAGA	Tetracycline
dfrA1_1	GGAATGGCCCTGATATTCCA	AGTCTTGCGTCCAACCAACAG	Trimethoprim
dfrG	TCAATCGGAAGAGCCTTACCTGA	TGGGCAAATACCTCATTCCATTCC	Trimethoprim
dfra17	CGGGAACGGCCCTGATATTCC	CGTGTTGCGACCGCATACTTTC	Trimethoprim
dfra21	TTGTTTCAACGCTGTCGCA	GGTTTCGGTTGAGACAAGCTC	Trimethoprim
dfrK	TGCTGCGATGGATAAGAACAG	CTTCCAGGTAATGCTCTTCCG	Trimethoprim
dfrA15	AGGCCGAAAGACTTTCGAGTC	TCACCTTCTGGCTCAATGTCG	Trimethoprim
aadA1_2	TGTACGGCTCCGCAGTG	CACGGAATGATGTCGTCGTG	Aminoglycoside

aadA2_3	CAATGACATTCTTGCGGGTATC	GACCTACCAAGGCAACGCTATG	Aminoglycoside
aadA_1	GTTGTGCACGACGACATCATT	GGCTCGAAGATACCTGCAAGAA	Aminoglycoside
aadA1_1	TGTACGGCTCCGCAGTG	CACGGAATGATGTCGTCGTG	Aminoglycoside
strB	GCTCGGTCGTGAGAACAATCT	CAATTTCGGTCGCCTGGTAGT	Aminoglycoside
ant6-ib	AGAACATCCGACAGCACGTTC	CCAACCTTCCATGAAATCATTCGC	Aminoglycoside
aac(6)-im	CGTGAGCATTATACAGAGCAATGG	CCATTTCCGTTCGTAGATATTGGC	Aminoglycoside
strA	CCGGTGGCATTTGAGAAAAA	GTGGCTCAACCTGCGAAAAG	Aminoglycoside
aadE	TACCTTATTGCCCTTGGAAGAGTTA	GGAACTATGTCCCTTTTAATTCTACAATCT	Aminoglycoside
aac(3)-iid_iia	CGATGGTCGCGGTTGGTC	TCGGCGTAGTGCAATGCG	Aminoglycoside
aph(2')-Ib	TGAGCAGTATCATAAGTTGAGTGAAAAG	GACAGAACAATCAATCTCTATGGAATG	Aminoglycoside
ant6-ia	TCGCCATGAGCTGCTGA	CCTATCATACTCCGGATAGGCATA	Aminoglycoside
aadA7	CACTCCGCGCCTTGGA	TGTGGCGGGCTCGAAG	Aminoglycoside
aph4-ib	GGGAACACCGTGCTCACC	GTTGGTCCCGTGCAGGTC	Aminoglycoside
aphA3_1	AAAAGCCCGAAGAGGAACTTG	CATCTTTCACAAAGATGTTGCTGTCT	Aminoglycoside
spcN	GCTATGTGCTGGTGGACTGG	GGAACCACTCGACGAACTCG	Aminoglycoside
aacC2	ACGGCATTCTCGATTGCTTT	CCGAGCTTCACGTAAGCATTT	Aminoglycoside
aph(3'')-ia	TAACAGCGATCGCGTATTTCG	TCCGACTCGTCCAACATCAATA	Aminoglycoside
str	AATGAGTTTTGGAGTGTCTCAACGTA	AATCAAAACCCCTATTAAAGCCAAT	Aminoglycoside
aadA9_1	CGCGGCAAGCCTATCTTG	CAAATCAGCGACCGCAGACT	Aminoglycoside
aac6-aph2	CCAAGAGCAATAAGGGCATACCAA	GCCACACTATCATAACCACTACCG	Aminoglycoside
aphA1/7	TGAACAAGTCTGGAAAGAAATGCA	CCTATTAATTTCCCCTCGTCAAAAA	Aminoglycoside
aadA5_2	ATCACGATCTTGCGATTTTGCT	CTGCGGATGGGCCTAGAAG	Aminoglycoside
sul2_2	TCATCTGCCAAACTCGTCGTTA	GTCAAAGAACGCCGCAATGT	Sulfonamide
folA_1	CGAGCAGTTCCTGCCAAAG	CCCAGTCATCCGGTTCATAATC	Sulfonamide
ermB_2	GAACACTAGGGTTGTTCTTGCA	CTGGAACATCTGTGGTATGGC	MLSB
lnuC	GGGTGTAGATGCTCTTCTTGGA	CTTTACCCGAAAGAGTTTCTACCG	MLSB
ermF	TCTGATGCCCGAAATGTTCAAG	TGAAGGACAATTGAACCTCCCA	MLSB
ermF_1	CAGCTTTGGTTGAACATTTACGAA	AAATTCCTAAAATCACAACCGACAA	MLSB
lnuA_1	TGACGCTCAACACACTCAAAAA	TTCATGCTTAAGTTCCATACGTGAA	MLSB
lmrA_1	TTCAGATGCAATGGCGTTTG	ATAATCGGGAACATAATGAGCATAACTAC	MLSB
ermX_2	TGATGACGGCTCAGTGG	GTGCACCAGCGCCTGA	MLSB
vatE_2	GACCGTCCTACCAGGCGTAA	TTGGATTGCCACCGACAATT	MLSB
msrC_1	TCAGACCGGATCGGTTGTC	CCTATTTTTTGGAGTCTTCTCTCTAATGTT	MLSB
mphB	CGCAGCGCTTGATCTTGTAG	TTACTGCATCCATACGCTGCTT	MLSB
ermT_1	GTTCACTAGCACTATTTTTAATGACAGAAGT	GAAGGGTGTCTTTTTAATACAATTAACGA	MLSB
ermA	TCGTTGAGAAGGGATTTGCGA	TTGCATGCTTCAAAGCCTGTC	MLSB
ereA	GATAATTCTGCTGGCGCACA	GCAGGCGTGGTCACAAC	MLSB
mefA	TAATTATCGCAGCAGCTGGTTC	GTTCCCAAACGGAGTATAAGAGTG	MLSB
mphA	TCAGCGGGATGATCGACTG	GAGGGCGTAGAGGGCGTA	MLSB
lnuB	GGATCGTTTACCAAAGGAGAAGG	AGCATAGCCTTCGTATCAGGAA	MLSB
erm36	GGCGGACCGACTTGCAT	TCTGCGTTGACGACGGTTAC	MLSB
msrE	CGGCAGATGGTCTGAGCTTAAA	CGCACTCTTCCTGCATAAAGGA	MLSB
blaTEM	CGCCGCATACACTATTCTCAG	GCTTCATTCAGCTCCGGTTC	Beta Lactam
pbp	CCGGTGCCATTGGTTTAGA	AAAATAGCCGCCCCAAGATT	Beta Lactam
blaCMY_3	CTGGCGCATACCTGGATTAC	GCCAGTTCAGCATCTCCCA	Beta Lactam
blaACC	CACACAGCTGATGGCTTATCTAAAA	AATAAACGCGATGGGTTCCA	Beta Lactam
blaACT	AAGCCGCTCAAGCTGGA	GCCATATCCTGCACGTTGG	Beta Lactam
cfxA	TCATTCCTCGTTCAAGTTTTCAGA	TGCAGCACCAAGAGGAGATGT	Beta Lactam
cepA	AGTTGCGCAGAACAGTCCTCTT	TCGTATCTTGCCCGTCGATAAT	Beta Lactam
blaSFO	CCGCCGCCATCCAGTA	GGGCCGCCAAGATGCT	Beta Lactam
blaOXY1	AAAGGTGACCGCATTCGC	CCAGCGTCAGCTTGCG	Beta Lactam
penA	AGACGGTAACGTATAACTTTTGAAAGA	GCGTGTAGCCGGCAATG	Beta Lactam
blaPSE	TTGTGACCTATTCCCCTGTAATAGAA	TGCGAAGCACGCATCATC	Beta Lactam
blaCTX-M	CGTACCGAGCCGACGTTAA	CAACCCAGGAAGCAGGCA	Beta Lactam
qnrB	GCGACGTTCAGTGGTTCAGA	GCTGCTCGCCAGTCGAA	Quinolone
qepA	GGGCATCGCGCTGTTC	GCGCATCGGTGAAGCC	Quinolone
cmlA_2	TAGGAAGCATCGGAACGTTGAT	CAGACCGAGCACGACTGTTG	Phenicol

floR	AACCCGCCCTCTGGATCA	GCCGTCGAGAAGAAGACGAA	Phenicol
catB2	GCTACTATTCCGGCTATTACCATG	GGGCTCCTCGTTCATGTAGA	Phenicol
cmxA	GCGATCGCCATCCTCTGT	TCGACACGGAGCCTTGGT	Phenicol
qacEDelta1_3	GTCGGTGTTGCTTATGCAGTCT	CAACCAGGCAATGGCTGTAA	Other
crAss64	TGTATAGATGCTGCTGCAACTGTACTC	CGTTGTTTTCATCTTTATCTTGTCCAT	Other
sat4	GAATGGGCAAAGCATAAAAACTTG	CCGATTTTGAAACCACAATTATGATA	Other
merA	GTGCCGTCCAAGATCATG	GGTGGAAGTCCAGTAGGGTGA	Other
vanC2	TGACTGTCGGTGCTTGTGA	GATAGAGCAGCTGAGCTTGTTC	Vancomycin
vanTC_2	ACAGTTGCCGCTGGTGAAG	CGTGGCTGGTCGATCAAAA	Vancomycin
vanRC4	AGTGCTTTGGCTTATCTCGAAAA	TCCGGCAGCATCACATCTAA	Vancomycin
vanA	GGGCTGTGAGGTCGGTTG	TTCAGTACAATGCGGCCGTTA	Vancomycin
vanC_2	CCTGCCACAATCGATCGTT	CGGCTTCATTCGGCTTGATA	Vancomycin
vanSC_2	ATCAACTGCGGGAGAAAAGTCT	TCCGCTGTTCCGCTTCTT	Vancomycin
vanTE	GTGGTGCCAAGGAAGTTGCT	CGTAGCCACCGCAAAAAAAT	Vancomycin

Table S2. Number of detected gene assays and their percentages for each group, overall and according with the two locations.

	**Detected Gene Assays**	**%**	**Detected Gene Assays**	**%**	**Detected Gene Assays**	**%**
	Overall		FreitaAMM		MontesinhoNP	
Aminoglycoside	23	24%	23	26%	11	24%
Tetracycline	19	20%	16	18%	10	22%
MLSB	18	19%	18	20%	8	18%
β-lactam	12	12%	10	11%	6	13%
Vancomycin	7	7%	6	7%	4	9%
Trimethoprim	6	6%	6	7%	0	0%
Phenicol	4	4%	3	3%	1	2%
Other	4	4%	4	4%	3	7%
Quinolone	2	2%	2	2%	1	2%
Sulfonamide	2	2%	2	2%	1	2%

Table S3. Average relative abundances for each group, overall and according with the two locations.

	**Average Relative Abundances**		
	Overall	FreitaAMM	MontesinhoNP
Trimethoprim	3.2E-03	3.2E-03	
Tetracycline	2.7E-03	2.6E-03	2.9E-03
Aminoglycoside	2.1E-03	2.8E-03	1.8E-04
β-lactam	2.1E-03	2.9E-03	4.4E-04
MLSB	1.9E-03	2.2E-03	5.8E-04
Sulfonamide	1.8E-03	2.3E-03	1.6E-04
Quinolone	1.1E-03	1.3E-03	2.1E-04
Phenicol	7.4E-04	8.6E-04	2.3E-05
Other	5.4E-04	7.7E-04	2.2E-05
Vancomycin	2.0E-04	4.2E-05	4.9E-04

Table S4. Percentages of resistant (according to the clinical breakpoints) and non-WT (according to the ECOFFs calculated using the NRI method) *E. coli* isolates for the tested antibiotics, overall and according with the two locations.

Antibiotic	Resistant	Non-WT	Resistant	Non-WT	Resistant	Non-WT
	Overall		FreitaAMM		MontesinhoNP	
Ampicillin	22%	19%	22%	19%	0%	0%
AMC	3%	0%	3%	0%	0%	0%
Cefoxitin	9%	0%	9%	0%	0%	0%
Cefotaxime	0%	0%	0%	0%	0%	0%
Ceftazidime	3%	0%	3%	0%	0%	0%
Aztreonam	0%	0%	0%	0%	0%	0%
Imipenem	0%	0%	0%	0%	0%	0%
Gentamicin	3%	0%	3%	0%	0%	0%
Amikacin	16%	0%	13%	0%	3%	0%
Tobramycin	3%	0%	0%	0%	3%	0%
Streptomycin	16%	9%	13%	9%	3%	0%
Nalidixic Acid	9%	9%	9%	9%	0%	0%
Ciprofloxacin	9%	44%	9%	31%	0%	13%
SXT	13%	13%	13%	13%	0%	0%
Tetracycline	19%	19%	16%	16%	3%	3%
Chloramphenicol	3%	3%	3%	3%	0%	0%
Nitrofurantoin	0%	3%	0%	3%	0%	0%

Table S5. Multidrug resistance phenotypes detected according to the clinical breakpoints.

Isolates		Resistance phenotype	Collection site
E. coli	RF12, RF13	AMP-S-NA-CIP-SXT-TE	FreitaAMM
E. coli	RF14	AMP-S-SXT-TE	FreitaAMM
E. coli	RF16	AMP-FOX-AK-TE	FreitaAMM
E. coli	RF17	AMP-AMC-FOX-CAZ-CN-AK-S-NA-CIP-SXT-TE-C	FreitaAMM
E. faecium	RF5	E-QDA-TE-TIG	MontesinhoNP

Table S6. Percentages of resistant (according to the clinical breakpoints) and non-WT (according to the ECOFFs calculated using the NRI method) *Enterococcus spp.* isolates for the tested antibiotics, overall and according with the two locations.

Antibiotic	Resistant	Non-WT	Resistant	Non-WT	Resistant	Non-WT
	Overall		FreitaAMM		MontesinhoNP	
Ampicillin	0%	0%	0%	0%	0%	0%
Chloramphenicol	0%	0%	0%	0%	0%	0%
Ciprofloxacin	0%	0%	0%	0%	0%	0%
Erythromycin	15%	24%	9%	12%	6%	12%
QDA	42%	0%	24%	0%	18%	0%
Tetracycline	18%	18%	15%	15%	3%	3%
Tigecycline	27%	15%	12%	6%	15%	9%
Gentamicin	0%	33%	0%	24%	0%	9%
Streptomycin	3%	3%	3%	3%	0%	0%
Teicoplanin	6%	0%	6%	0%	0%	0%
Vancomycin	0%	0%	0%	0%	0%	0%

References

1. Stedtfeld, R.D.; Guo, X.; Stedtfeld, T.M.; Sheng, H.; Williams, M.R.; Hauschild, K.; Gunturu, S.; Tift, L.; Wang, F.; Howe, A.; et al. Primer Set 2.0 for Highly Parallel QPCR Array Targeting Antibiotic Resistance Genes and Mobile Genetic Elements. *FEMS Microbiol. Ecol.* **2018**, *94*, fiy130. https://doi.org/10.1093/femsec/fiy130.
